# Hospital Length of Stay and Associated Factors in Patients with Osteoarthritis from Germany: A Cross-Sectional Study

**DOI:** 10.3390/jcm13092628

**Published:** 2024-04-30

**Authors:** Karel Kostev, Razak M. Gyasi, Marcel Konrad, Dong Keon Yon, Louis Jacob

**Affiliations:** 1 Epidemiology, IQVIA, Unterschweinstiege 2-14, 60549 Frankfurt am Main, Germany; 2 University Clinic, Philipps-University, 35037 Marburg, Germany; 3 African Population and Health Research Center, Nairobi 00100, Kenya; 4 National Centre for Naturopathic Medicine, Faculty of Health, Southern Cross University, Lismore, NSW 2480, Australia; 5 Department of Health and Social, FOM University of Applied Sciences for Economics and Management, 45127 Essen, Germany; 6 Center for Digital Health, Medical Science Research Institute, Kyung Hee University College of Medicine, Seoul 02447, Republic of Korea; 7 Department of Regulatory Science, Kyung Hee University, Seoul 02447, Republic of Korea; 8 Department of Pediatrics, Kyung Hee University Medical Center, Kyung Hee University College of Medicine, Seoul 02447, Republic of Korea; 9 Research and Development Unit, Parc Sanitari Sant Joan de Déu, CIBERSAM, ISCIII, Dr. Antoni Pujadas, 42, 08830 Sant Boi de Llobregat, Spain; 10 AP-HP, Université Paris Cité, Lariboisière-Fernand Widal Hospital, Department of Physical Medicine and Rehabilitation, 75010 Paris, France; 11 Université Paris Cité, Inserm U1153, Epidemiology of Ageing and Neurodegenerative Diseases (EpiAgeing), 75010 Paris, France

**Keywords:** associated factors, epidemiology, geriatric rehabilitation, Germany, hospital length of stay, osteoarthritis

## Abstract

**Objective**: There is a scarcity of data on hospital length of stay (LOS) in the osteoarthritis population. Therefore, this study aimed to investigate hospital LOS and associated factors in patients with osteoarthritis from Germany. **Methods**: The present cross-sectional study included patients hospitalized for osteoarthritis in one of fourteen hospitals in Germany between 2018 and 2023 (hospital database; IQVIA). The study outcome was the duration of hospital stay in days. Study covariables included age, sex, hospital department, osteoarthritis type, co-diagnosis, and hospitalization-related procedure. Associations between covariables and hospital LOS were analyzed using hierarchical linear regression models. **Results**: There were 8770 patients included in the study (mean [standard deviation] age 68.7 [10.8] years; 60.2% women). The mean (standard deviation) hospital LOS was 8.5 (5.0) days. Factors positively and significantly associated with hospital LOS were older age, female sex, orthopedic surgery and other medical specialty departments (compared with other surgery departments), knee and other and unspecified osteoarthritis (compared with hip osteoarthritis), multiple co-diagnoses (e.g., acute posthemorrhagic anemia, other disorders of fluid, electrolyte, and acid–base balance, and disorders of purine and pyrimidine metabolism), and several hospitalization-related procedures (i.e., geriatric rehabilitation, hip arthroplasty, and knee arthroplasty). **Conclusions**: The mean hospital LOS was higher than eight days in this osteoarthritis population from Germany, with a spectrum of demographic, clinical, and hospitalization-related factors associated with this hospital LOS. In this context, interventions are needed to reduce the LOS of hospitalizations for osteoarthritis in Germany.

## 1. Introduction

Osteoarthritis is a chronic condition of the joint characterized by several symptoms, such as pain, stiffness, and joint limitation [1]. Common pathological changes involved in osteoarthritis include cartilage degradation, local inflammation, and the formation of osteophytes. There were 595 million people with osteoarthritis in the world in 2020, which corresponded to a global prevalence of 7.6% [2]. The number of adults affected by osteoarthritis will increase in the following decades, with this increasing trend being partially explained by the aging of the worldwide population. There is a significant association between osteoarthritis and increased mortality, and this relationship likely involves, at least partially, cardiovascular diseases [3]. In addition, osteoarthritis is associated with several physical (e.g., diabetes [4] and hypertension [5]) and psychiatric chronic conditions (e.g., anxiety [6] and depression [7]). Finally, individuals with osteoarthritis display lower health-related quality of life compared with the general population [8].

In addition to these effects on health, osteoarthritis involves major direct and indirect costs. Direct costs are defined as costs related to the management and treatment of a disease, whereas indirect costs correspond to broader costs due to work absenteeism and reduced work productivity [9]. For example, a systematic review of 32 cost-of-illness studies found that the annual cost of lower-limb osteoarthritis per patient ranged from EUR 700 to EUR 12,000, with the direct costs being estimated at EUR 500–10,900 and indirect costs at EUR 200–12,300 [10]. Direct costs include hospitalization-related costs, and these costs increase with hospital length of stay (LOS) [11]. Multiple studies have analyzed hospital LOS in people with osteoarthritis [12,13,14,15,16,17,18]. Although these studies have advanced the field, all of them focused on LOS following arthroplasty, and little is known about LOS in osteoarthritis patients hospitalized for conservative treatments (e.g., physical therapy, occupational therapy, and intra-articular corticosteroid injections). Taking this limitation into consideration, more data on hospital LOS in the osteoarthritis population are warranted.

Therefore, the goal of this retrospective study was to analyze hospital LOS and identify associated factors in patients with osteoarthritis from 14 hospitals in Germany. Given that osteoarthritis is a frequent disorder in the German population [19], it is critical to better understand the drivers of prolonged hospitalization in people with osteoarthritis in order to improve the allocation of economic resources for the management and treatment of this chronic condition in this country.

## 2. Method

### 2.1. Guidelines

The study adheres to the STROBE guidelines.

### 2.2. Database

The present cross-sectional study was based on the data from the hospital database (company: IQVIA, Frankfurt, Germany). The participating hospitals are maximum care (i.e., large institutions with usually more than 1000 beds), primary care, specialized, standard care, and university hospitals. Data collected by IQVIA correspond to standardized information transmitted by hospitals to the Institute for the Hospital Remuneration System (InEK) in accordance with Section 21 of the German Hospital Compensation Act (*Krankenhausentgeltgesetz* [KHEntgG] in German). For each hospitalization, there are data on primary diagnoses, secondary diagnoses, and procedures performed during the hospitalization. Diagnoses are coded using the International Classification of Diseases, 10th revision (ICD-10), while hospitalization-related procedures are coded using the OPS classification (*Operationen- und Prozedurenschlüssel* in German). Finally, these routine data are sent to IQVIA on a regular basis and in an anonymized format.

### 2.3. Study Population

The study included patients hospitalized for osteoarthritis (ICD-10: M15-M19) from 14 hospitals across Germany between November 2018 and October 2023. If there were several hospitalizations for the same individual during the study period, only the first hospitalization was included in the analyses.

### 2.4. Study Outcome

The outcome of the study was the duration of hospital stay in days.

### 2.5. Study Covariates

The study covariates were age (in years; continuous and categorical variable [i.e., ≤50, 51–60, 61–70, 71–80, and >80]), sex (i.e., female and male), hospital department (i.e., orthopedic surgery, other surgery, geriatric, and other medical specialty), osteoarthritis type (i.e., osteoarthritis of hip [ICD-10 code: M16], osteoarthritis of knee [ICD-10 code: M17], and other and unspecified osteoarthritis [ICD-10 code: M19]), and co-diagnosis (i.e., essential hypertension [ICD-10 code: I10], overweight and obesity [ICD-10 code: E66], thyroid gland disorders [ICD-10 codes: E00-E07], lipid metabolism disorders [ICD-10 code: E78], diabetes mellitus [ICD-10 codes: E10-E14], coronary heart disease [ICD-10 code: I25], other disorders of fluid, electrolyte, and acid–base balance [ICD-10 code: E87], atrial fibrillation and flutter [ICD-10 code: I48], disorders of purine and pyrimidine metabolism [ICD-10 code: E79], depression [ICD-10 codes: F32 and F33], sleep disorders [ICD-10 code: G47], and acute posthemorrhagic anemia [ICD-10 code: D62]), and hospitalization-related procedure (i.e., physical therapy, geriatric rehabilitation, hip arthroplasty, and knee arthroplasty). The co-diagnoses of interest were those found in at least 5% of the population.

### 2.6. Statistical Analyses

Covariates were described in the population using absolute numbers (percentages), except continuous age, which was described using the mean (standard deviation). Moreover, the mean (standard deviation) hospital LOS was studied in the overall sample and by age, sex, hospital department, and osteoarthritis type. Finally, the associations between the covariates (independent variables) and hospital LOS (dependent variable) were analyzed with hierarchical linear regression models. The first model was adjusted for age, sex, hospital department, and osteoarthritis type. The second model was adjusted for the same variables as in the first model and co-diagnosis. The third model was adjusted for the same variables as in the second model and hospitalization-related procedure. A sensitivity analysis was conducted by hospitalization-related procedure (i.e., physical therapy or geriatric rehabilitation [without arthroplasty], hip arthroplasty, and knee arthroplasty), as factors associated with LOS in osteoarthritis may vary with the type of procedure. The results of the linear regressions are displayed as ß coefficients (differences in days) with *p*-values. There were no missing data in any of the variables included in the analyses. Two-sided *p*-values lower than 0.050 were considered statistically significant. All analyses were performed using SAS version 9.4 (SAS Institute, Cary, NC, USA).

## 3. Results

**Figure 1:**
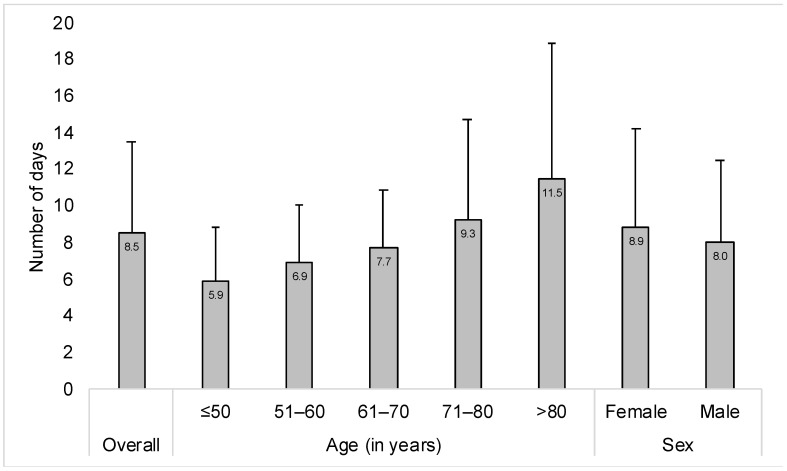
Hospital length of stay in the overall population and by age and sex.

**Figure 2:**
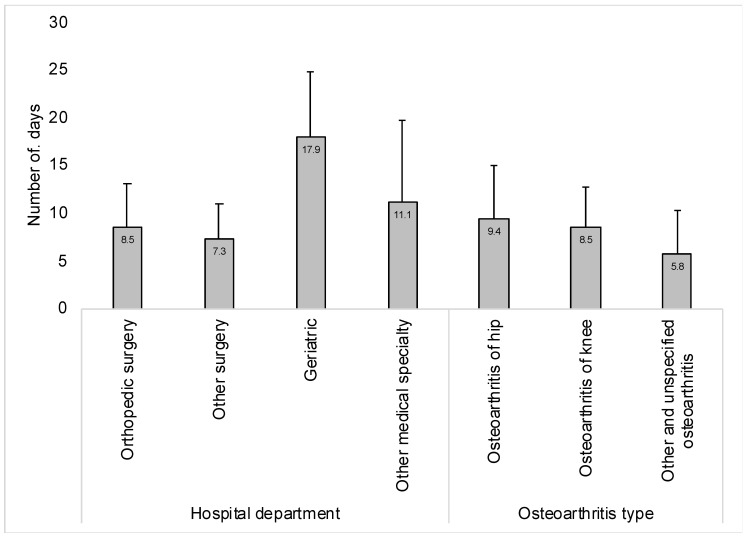
Hospital length of stay by hospital department and osteoarthritis type.

This study included 8770 patients hospitalized for osteoarthritis. The characteristics of the study population are displayed in Table 1. The mean (standard deviation) age was 68.7 (10.8) years, while there were 60.2% of women. The majority of the sample was hospitalized in orthopedic surgery (i.e., 42.1%) or other surgery departments (i.e., 48.7%). The most frequent osteoarthritis type was knee osteoarthritis (i.e., 46.0%). Finally, the three most common co-diagnoses were essential hypertension (i.e., 61.0%), overweight and obesity (i.e., 25.6%), and thyroid gland disorders (i.e., 21.6%). Figure 1 and Figure 2 show the mean (standard deviation) hospital LOS in the overall population and by age, sex, hospital department, and osteoarthritis type. The mean (standard deviation) hospital LOS was 8.5 (5.0) days in the whole sample and increased from 5.9 (3.0) days in people aged ≤ 50 years to 11.5 (7.4) days in those aged >80 years. In terms of sex, the figure was slightly higher in women (i.e., 8.9 [5.3] days) than in men (i.e., 8.0 [4.5] days). In addition, the mean (standard deviation) LOS was particularly high in individuals hospitalized in geriatric departments (i.e., 17.9 [6.8] days). At the same time, the figure was higher in participants with hip (i.e., 9.4 [5.6] days) or knee osteoarthritis (i.e., 8.5 [4.3] days) compared with those with other and unspecified osteoarthritis (i.e., 5.8 [4.4] days). Finally, the results of the hierarchical linear regression models are displayed in Table 2. In the model including all covariates, age was positively and significantly associated with hospital LOS, with ß coefficients ranging from 0.44 days in the age group 51–60 years to 1.99 days in the age group >80 years (reference: ≤50 years). Moreover, men had shorter hospital LOS compared with women (i.e., ß coefficient = −0.32 days). People hospitalized in orthopedic surgery (i.e., ß coefficient = 0.27 days) and other medical specialty departments (i.e., ß coefficient = 2.57 days) were more likely to have prolonged hospital LOS than those hospitalized in other surgery departments. In terms of osteoarthritis type, knee osteoarthritis (ß coefficient = 0.80 days) and other and unspecified osteoarthritis (i.e., ß coefficient = 0.68 days) were risk factors for longer hospital LOS (reference: hip osteoarthritis). In addition, there were nine co-diagnoses significantly associated with hospital LOS, and effect sizes were the strongest for acute posthemorrhagic anemia (i.e., ß coefficient = 3.57 days), other disorders of fluid, electrolyte, and acid–base balance (i.e., ß coefficient = 1.21 days), and disorders of purine and pyrimidine metabolism (i.e., ß coefficient = 0.59 days). Finally, undergoing geriatric rehabilitation (i.e., ß coefficient = 11.69 days), hip arthroplasty (i.e., ß coefficient = 4.47 days), or knee arthroplasty (i.e., ß coefficient = 3.52 days) was associated with an increase in hospital LOS, whereas there was a negative and significant relationship between physical therapy and hospital LOS (i.e., ß coefficient = −2.09 days). Similar findings were obtained in the sensitivity analysis conducted by hospitalization-related procedures, although some of the associations did not reach statistical significance (Table 3).

## 4. Discussion

### 4.1. Main Findings

In this study of more than 8700 adults hospitalized for osteoarthritis in Germany, the mean hospital LOS was higher than eight days. Increased hospital LOS was significantly associated with older age, female sex, hospitalization in orthopedic surgery or other medical specialty departments (compared with hospitalization in other surgery departments), knee and other and unspecified osteoarthritis (compared with hip osteoarthritis), nine co-diagnoses (the diagnoses with the strongest effect sizes being acute posthemorrhagic anemia, other disorders of fluid, electrolyte, and acid–base balance, and disorders of purine and pyrimidine metabolism), and three hospitalization-related procedures (i.e., geriatric rehabilitation, hip arthroplasty, and knee arthroplasty). In contrast, compared with no physical therapy, physical therapy was associated with shorter hospital LOS.

### 4.2. Interpretation of the Findings

The study results are a critical contribution to the scientific literature, which exclusively focused on hospital LOS in osteoarthritis patients undergoing arthroplasty of the hip or the knee. It is interesting to note that the hospital LOS reported in the present study is higher than the figures previously reported. For example, it was observed, in a sample of 103,511 osteoarthritis patients with primary hip or knee replacement in New Zealand between 2005 and 2017, that the mean hospital LOS was between 4.7 and 5.4 days [17]. The discrepancy between the findings of the present research and prior results may be explained by the fact that this study included osteoarthritis patients receiving surgical treatment and those receiving medical treatment, such as geriatric rehabilitation, which may require a longer hospital LOS. In addition, these bodies of research were conducted in different countries and regions of the world, and there may be substantial differences in the healthcare systems and the management of osteoarthritis.

This study also identified several demographic, clinical, and hospitalization-related factors significantly associated with hospital LOS. Older adults had longer hospital LOS than their younger counterparts. This result could be related to the fact that the odds of complications associated with the management and treatment of osteoarthritis increase with age [20]. In addition, the female sex was a risk factor for prolonged hospital LOS compared with the male sex. This result should be interpreted with caution, as the male sex has been found to be positively associated with multiple early unfavorable outcomes following surgical treatment for osteoarthritis [21,22,23] That being said, some of these studies did observe at the same time that the hospital LOS was longer in female than male patients [21,22], and this difference may be related to the more frequent use of blood transfusion in women, which may require prolonged hospitalization.

In terms of hospital departments, being hospitalized in orthopedic surgery or other medical specialty departments was positively and significantly associated with hospital LOS compared with being hospitalized in other surgery departments. It is likely that individuals with complex forms of osteoarthritis are more likely to be treated in specialized surgery departments than those with less complex forms. Regarding other medical specialty departments, such as rheumatology departments, one hypothesis is that multiple corticosteroid injections may be performed during a single hospitalization, and these infiltrations may delay the discharge from the hospital. Moreover, patients hospitalized in these departments may display comorbidities (e.g., diabetes [24] and hypertension [25]), and the management and treatment of these conditions may postpone discharge from the hospital. Furthermore, knee and other and unspecified osteoarthritis were positively associated with hospital LOS compared with hip osteoarthritis. The previous literature has also reported that hospital LOS is slightly higher for knee arthroplasty than hip arthroplasty [26], and better immediate post-operative recovery for hip arthroplasty compared with knee arthroplasty could be an underlying factor for this difference [27]. The other and unspecified osteoarthritis group may include a substantial proportion of people with osteoarthritis of the neck, thoracic back, and lower back, which may warrant extensive medical treatment.

There was a positive and significant relationship between several physical conditions and hospital LOS. Of particular importance, acute posthemorrhagic anemia was associated with an increase of more than three days in hospital LOS. Anemia is a frequent complication after arthroplasty, with blood loss estimated between 250 and 750 mL for a primary total hip arthroplasty [28]. People with anemia may receive a blood transfusion, and multiple blood tests may be needed to assess hemoglobin levels prior to hospital discharge. There was also a relationship between other disorders of fluid, electrolyte, and acid–base balance with hospital LOS, and the effect size of the association was higher than one day. This result should be interpreted cautiously, as previous research found no correlation between post-operative hyponatremia and hospital LOS [29] However, the present study did not focus on hyponatremia and also included other types of electrolyte disorders. The conditions with the third strongest effect size were disorders of purine and pyrimidine metabolism. This family of disorders includes an extensive range of diseases, and gout and hyperuricemia belong to this family. Interestingly, gout has been identified as a risk factor for longer LOS in people hospitalized for heart failure exacerbation [30] This relationship may underline that the management of gout is complex and may involve the treatment of gout flares, the initiation of long-term medications, and patient education [31]. Finally, multiple diseases were associated with a more modest but still statistically significant increase in hospital LOS, with the clinical relevance of these associations being unclear.

Several procedures were positively associated with hospital LOS. The effect size was the highest for geriatric rehabilitation, with a beta coefficient of around 11.7 days. This rehabilitation is multidisciplinary and combines the expertise of different health professionals (e.g., geriatricians, occupational therapists, and physical therapists). In this context, geriatric rehabilitation likely results in hospitalizations lasting a few weeks. Hip and knee arthroplasty also increased hospital LOS, with the respective effect sizes being 4.5 and 3.5 days. This observation underlines that the implementation of fast-track surgery is slightly lower in Germany than in other European countries. Reasons for the insufficient development of fast-track programs in this country could include the underdeveloped cooperation between different medical and surgical specialties and healthcare reluctance to change [32]. Finally, physical therapy was associated with shorter hospital LOS compared with no physical therapy. Although this result should be apprehended carefully, individuals offered physical therapy may be those with osteoarthritis not severe enough to require surgery and may be those of middle age not undergoing geriatric rehabilitation.

### 4.3. Clinical Implications and Directions for Future Research

Based on the present findings, hospital LOS could be a critical contributor to the direct costs associated with the management and treatment of osteoarthritis in Germany. Measures should be taken to reduce LOS in people hospitalized for osteoarthritis. From a medical perspective, it is essential to identify individuals with comorbidities that could prevent early hospital discharge. From a surgical perspective, there is a need to facilitate the implementation of fast-track programs. In terms of future research, more data from other countries are warranted before any firm conclusion is drawn. In addition, further studies should analyze the effects of other sociodemographic factors, living status, and health behaviors on hospital LOS in people with osteoarthritis.

### 4.4. Strengths and Limitations

The strengths of the study are the sample size, the number of participating hospitals, and the large panel of collected data. Nonetheless, the study also displays several limitations that need to be acknowledged. First, there were no data on the underlying reasons for delayed hospital discharge in patients with prolonged hospital LOS. Second, patients may have been transferred to hospitals not participating in the study, and this information was not documented, potentially underestimating hospital LOS. Third, health behaviors were not available, and it was, therefore, not possible to analyze their effects on hospital LOS. Fourth, more details about hip and knee arthroplasty, as well as on the components of geriatric rehabilitation, would have allowed more detailed analyses.

## 5. Conclusions

This study of more than 8700 people with osteoarthritis from 14 hospitals in Germany revealed that the hospital LOS was higher than eight days. Several demographic, clinical, and hospitalization-related procedures were statistically associated with hospital LOS. Finally, based on these findings, measures should be taken to reduce the LOS related to hospitalizations for osteoarthritis, and more data are needed to corroborate or invalidate the results in other countries.

## Figures and Tables

**Table 1:** Characteristics of the study population.

Variable	Population (N = 8770)
*Age (in years)*	
Mean (standard deviation)	68.7 (10.8)
≤50	391 (4.5)
51–60	1659 (18.9)
61–70	2697 (30.8)
71–80	2670 (30.4)
>80	1353 (15.4)
*Sex*	
Female	5277 (60.2)
Male	3493 (39.8)
*Hospital department*	
Orthopedic surgery	3691 (42.1)
Other surgery	4271 (48.7)
Geriatric	458 (5.2)
Other medical specialty	350 (4.0)
*Osteoarthritis type*	
Osteoarthritis of hip	3700 (42.2)
Osteoarthritis of knee	4037 (46.0)
Other and unspecified osteoarthritis	1033 (11.8)
*Co-diagnosis*	
Essential hypertension	5352 (61.0)
Overweight and obesity	2248 (25.6)
Thyroid gland disorders	1894 (21.6)
Lipid metabolism disorders	1572 (17.9)
Diabetes mellitus	1335 (15.2)
Coronary heart disease	732 (8.4)
Other disorders of fluid, electrolyte, and acid–base balance	722 (8.2)
Atrial fibrillation and flutter	677 (7.7)
Disorders of purine and pyrimidine metabolism	555 (6.3)
Depression	533 (6.1)
Sleep disorders	497 (5.7)
Acute posthemorrhagic anemia	442 (5.0)
*Hospitalization-related procedure*	
Physical therapy	3113 (35.5)
Geriatric rehabilitation	507 (5.8)
Hip arthroplasty	3290 (37.5)
Knee arthroplasty	3608 (41.1)

Data are absolute numbers (percentages) unless otherwise specified.

**Table 2:** Association between demographic and clinical variables and length of stay in patients hospitalized for osteoarthritis (adjusted linear regression models).

Variable	Model 1 ^1^		Model 2 ^2^		Model 3 ^3^	
	ß Coefficient (Difference in Days)	*p*-Value	ß Coefficient (Difference in Days)	*p*-Value	ß Coefficient (Difference in Days)	*p*-Value
*Age (in years)*						
≤50	Reference		Reference		Reference	
51–60	0.71	0.003	0.56	0.014	0.44	0.031
61–70	1.24	<0.001	0.96	<0.001	0.85	<0.001
71–80	2.16	<0.001	1.61	<0.001	1.49	<0.001
>80	2.87	<0.001	2.10	<0.001	1.99	<0.001
*Sex*						
Female	Reference		Reference		Reference	
Male	−0.38	<0.001	−0.38	<0.001	−0.32	<0.001
*Hospital department*						
Other surgery	Reference		Reference		Reference	
Orthopedic surgery	1.46	<0.001	1.43	<0.001	0.27	0.016
Geriatric	9.46	<0.001	9.31	<0.001	0.43	0.307
Other medical specialty	5.12	<0.001	4.91	<0.001	2.57	<0.001
*Osteoarthritis type*						
Osteoarthritis of hip	Reference		Reference		Reference	
Osteoarthritis of knee	−0.28	0.003	−0.17	0.067	0.80	0.002
Other and unspecified osteoarthritis	−3.58	<0.001	−3.20	<0.001	0.68	0.008
*Co-diagnosis* ^4^						
Essential hypertension			0.10	0.314	0.03	0.726
Overweight and obesity			0.24	0.024	0.53	<0.001
Thyroid gland disorders			0.20	0.071	0.24	0.015
Lipid metabolism disorders			0.12	0.316	0.03	0.758
Diabetes mellitus			0.34	0.006	0.28	0.015
Coronary heart disease			0.51	0.002	0.54	<0.001
Other disorders of fluid, electrolyte, and acid–base balance			1.48	<0.001	1.21	<0.001
Atrial fibrillation and flutter			0.42	0.012	0.47	0.003
Disorders of purine and pyrimidine metabolism			0.87	<0.001	0.59	<0.001
Depression			0.48	0.009	0.32	0.057
Sleep disorders			0.70	<0.001	0.58	<0.001
Acute posthemorrhagic anemia			4.23	<0.001	3.57	<0.001
*Hospitalization-related procedure*						
Physical therapy					−2.09	<0.001
Geriatric rehabilitation					11.69	<0.001
Hip arthroplasty					4.47	<0.001
Knee arthroplasty					3.52	<0.001

^1^ Model 1 was adjusted for age, sex, hospital department, and osteoarthritis type. ^2^ Model 2 was adjusted for age, sex, hospital department, osteoarthritis type, and co-diagnosis. ^3^ Model 3 was adjusted for age, sex, hospital department, osteoarthritis type, co-diagnosis, and procedure. ^4^ The reference is the absence of the co-diagnosis of interest.

**Table 3:** Association between demographic and clinical variables and length of hospital stay in patients hospitalized for osteoarthritis by hospitalization-related procedure (adjusted linear regression models).

	Physical Therapy or Geriatric Rehabilitation (without Arthroplasty) (N = 457)		Hip Arthroplasty (N = 3290)		Knee Arthroplasty (N = 3608)	
Variable	ß Coefficient (Difference in Days)	*p*-Value	ß Coefficient (Difference in Days)	*p*-Value	ß Coefficient (Difference in Days)	*p*-Value
*Age (in years)*						
≤50	Reference		Reference		Reference	
51–60	0.12	0.690	0.17	0.680	−0.02	0.945
61–70	0.26	0.351	0.32	0.419	0.17	0.583
71–80	0.72	0.014	1.02	0.011	0.51	0.095
>80	1.13	<0.001	1.83	<0.001	1.35	<0.001
*Sex*						
*Female*	Reference		Reference		Reference	
*Male*	−0.12	0.239	−0.40	0.010	−0.39	<0.001
*Hospital department*						
Other surgery	Reference		Reference		Reference	
Orthopedic surgery	2.65	<0.001	1.28	<0.001	1.30	<0.001
Geriatric	10.0	<0.001	16.44	<0.001	18.33	<0.001
Other medical specialty	9.99	<0.001	13.28	<0.001	9.59	<0.001
*Osteoarthritis type*						
Osteoarthritis of hip	Reference		-		-	
Osteoarthritis of knee	−0.01	0.947	-		-	
Other and unspecified osteoarthritis	−0.80	0.116	-		-	
*Co-diagnosis*						
Essential hypertension	0.05	0.647	0.13	0.396	0.26	0.196
Overweight and obesity	0.30	0.006	0.30	0.092	−0.34	0.003
Thyroid gland disorders	0.04	0.760	−0.07	0.699	0.00	0.989
Lipid metabolism disorders	−0.17	0.199	0.10	0.637	0.03	0.801
Diabetes mellitus	0.36	0.010	0.45	0.042	0.25	0.080
Coronary heart disease	0.18	0.322	0.50	0.083	0.26	0.196
Other disorders of fluid, electrolyte, and acid–base balance	0.77	<0.001	1.18	<0.001	0.83	<0.001
Atrial fibrillation and flutter	0.31	0.119	0.75	0.012	0.35	0.109
Disorders of purine and pyrimidine metabolism	0.41	0.038	0.69	0.029	0.87	<0.001
Depression	0.14	0.469	0.31	0.356	−0.05	0.811
Sleep disorders	0.30	0.137	0.73	0.021	0.35	0.109
Acute posthemorrhagic anemia	2.65	<0.001	3.69	<0.001	2.78	<0.001

Models were adjusted for age, sex, hospital department, osteoarthritis type (for physical therapy or geriatric rehabilitation [without arthroplasty] only), and co-diagnosis.

## Data Availability

The data and the code used for this study are available from the corresponding authors upon reasonable request.
